# Metaviromic profiling of mosquito excreta using superhydrophobic collection devices expands the known RNA virome of North America

**DOI:** 10.1128/spectrum.00938-26

**Published:** 2026-07-28

**Authors:** Dana C. Price, Fiona L. Bezhani, Zhuolun Meng, Margherita Porfirio-LaStrapes, Nicole E. Wagner, Mehdi Javanmard, Taewon Han, Miranda M. Barnes

**Affiliations:** 1 Department of Entomology, Center for Vector Biology, Rutgers, The State University 242612, New Brunswick, New Jersey, USA; 2 Department of Electrical and Computer Engineering, Rutgers, The State University, Piscataway, New Jersey, USA; 3 Department of Environmental Sciences, Rutgers, The State University 242612, New Brunswick, New Jersey, USA; National Microbiology Laboratory, Winnipeg, Manitoba, Canada

**Keywords:** viruses, metaviromics, vector biology, mosquito, public health, metagenomics, *Culex pipiens*, vector-borne diseases, molecular epidemiology, surveillance studies

## Abstract

Nearly 30% of emerging infectious disease events worldwide are transmitted by arthropod vectors, and this proportion continues to rise. Rapid and accurate detection is critical for directing vector control interventions, thereby reducing the likelihood of widespread transmission. Surveillance of infected mosquitoes can provide an early warning of impending human infection; however, conventional virus testing relies on processing large pools of mosquitoes and requires labor-intensive pre-processing. During rapidly developing epidemic or panzootic events, these delays may limit the effectiveness of public health responses. Mosquito excreta has recently emerged as a promising alternative substrate for pathogen detection. Sugar-fed mosquitoes regularly excrete gut contents, offering a rich source of nucleic acids. In this study, we developed and applied custom superhydrophobic excreta-collection funnels that efficiently aggregate excreta produced by field-collected *Culex* mosquitoes into attached microcentrifuge tubes. Shotgun metagenomic sequencing of this material revealed a diverse RNA virome, including both globally distributed viruses and those reported here for the first time from the Americas. Beyond virus detection, additional analyses enabled confirmation of host mosquito species and identification of trypanosomatid parasites, demonstrating the broader utility of mosquito excreta for integrated surveillance. We anticipate that methods and devices of this type will become valuable components of vector surveillance programs, particularly in remote or resource-limited settings where repeated collections are challenging. Overall, our findings highlight the potential of excreta-based monitoring to improve early detection of emerging or unknown pathogens of One Health importance, refine our understanding of mosquito virome biogeography, and facilitate the discovery of previously undescribed viruses.

**IMPORTANCE:**

Many infectious diseases that affect people and animals are spread by mosquitoes and other biting insects, and the number of these outbreaks is increasing. Detecting pathogens in mosquito populations early can provide a critical warning before human cases begin, allowing health officials to act quickly. However, traditional surveillance requires collecting and processing large numbers of mosquitoes, which can be slow and labor-intensive during fast-moving outbreaks. Here we demonstrate a simpler approach: testing mosquito waste. When mosquitoes feed on sugar, they excrete material that contains genetic traces of viruses and other organisms. Using specially designed collection devices and modern genetic sequencing, we show that mosquito excreta can reveal a wide range of viruses and parasites while also identifying the mosquito species present. This method could make disease surveillance faster and more practical in remote or resource-limited settings, improving our ability to detect emerging pathogens that threaten human, animal, and environmental health.

## INTRODUCTION

The global vector-borne disease landscape is changing quickly. Nearly 30% of global emerging infectious disease events are vector-borne, and that share has only increased over time (1). Recent outbreaks of mosquito-vectored arboviruses–including Dengue, Chikungunya, and Zika viruses–have garnered significant attention and raised concerns regarding what will emerge next and where (2, 3). Factors responsible for shifting disease dynamics, such as land development, trade intensification, human migration, increases in population density, and climate change, show no signs of subsiding and are expected to promote future epidemics (4, 5). Recent international pandemics have highlighted weaknesses in responses to global health crises. In a June 2020 white paper “Preparing for the Next Pandemic,” the United States Senate Committee on Health, Education, Labor and Pensions Chairman outlined five identified gaps for Congress to address in the coming years; the second of these was “Disease Surveillance—Expand Ability to Detect, Identify, Model, and Track Emerging Infectious Diseases” (6), emphasizing that effective surveillance of both vectors and pathogens is critical to monitoring current outbreaks and assessing the potential for future outbreaks before they occur (7). As a result, the National Institute of Allergy and Infectious Diseases has allotted significant funding to studying designated “prototype pathogens” (representative viruses from families known to infect humans), developing medical countermeasures against some of the most likely threats, and expanding global pathogen monitoring efforts (8).

Early detection ensures that vector control efforts are applied to the right locations to avert an epidemic. The most common monitoring strategies include reporting and tracking of human cases, testing for serological conversion of sentinel hosts, and assessing the rate at which vectors are infected in the field (9–11). These methods all have advantages and disadvantages: waiting for the detection of human cases misses a critical window allowing for early intervention, while sentinel animals require care and maintenance and could potentially act as amplification reservoirs (11). Surveillance and detection of infected mosquitoes can serve as an early predictor of impending human infection (12); however, virus testing does not commonly occur until the insects have been appropriately identified and pooled. Large quantities of mosquitoes can require extended pre-processing steps, the bulk of which need to occur on a cold chain to prevent RNA degradation (13). Even though this is a standard protocol in the vector surveillance toolkit employed by health agencies and mosquito control programs, in emergent epi- and panzootic outbreaks, it may not provide information quickly enough for threat detection and response to be most effective.

Standard surveillance devices (e.g., CDC Light Trap, CDC Gravid Trap, Biogents Sentinel [BG-Sentinel] trap) are well-suited to basic mosquito collection, but their confined chambers and fans result in specimen morbidity and mortality, as well as a loss of nucleic acid integrity (14, 15). Relatively novel strategies employ CO_2_-baited mosquito traps that house sugar-soaked nucleic acid preservation cards (Flinders Technology Associates; FTA [GE Healthcare]) to capture and preserve viral RNA for remote arbovirus surveillance (11, 16, 17). As infected mosquitoes probe the sugar, they expectorate viruses and nucleic acids onto the cards. These compounds are immediately deactivated by the proprietary FTA reagent, which mitigates biosafety hazards from handling viable pathogens and protects the RNA from degradation at room temperature for at least 28 days (11, 17, 18). This method has many advantages: there is no requirement for mosquito sorting, ID, or pooling; cards can be easily collected and stored without upholding a cold chain; and positive cards indicate the presence of the pathogen in salivary glands, suggesting disseminated infection. FTA cards have been used to successfully detect viral transmission in populations more quickly than strategies employing mosquito pools or sentinel animals (11, 19, 20).

The success of this non-destructive means to survey mosquito populations suggests that mosquito excreta/feces may represent a useful source of template material for virus testing, as sugar-engorged mosquitoes frequently void their gut contents (21) and produce a greater volume of excreta (~1.5 µL) than they do saliva (<5 nL) (22). Molecular xenomonitoring—the detection of human or animal parasitic DNA or RNA within hematophagous insects—has demonstrated that mosquitoes with a disseminated arboviral infection excrete viruses and/or parasites with sufficient template volume for detection of Dengue (23, 24), West Nile, and Ross River viruses (25, 26), as well as DNA corresponding to many human filarial and malaria parasites and trypanosomes when sampled in laboratory conditions (26–28). Intriguingly, concentrations of Dengue virus have been found to be higher in excreta than in saliva (29), suggesting this material may in some cases prove more useful for obtaining high-yield molecular data.

Excreta-sampling trap devices tested in the field typically use a large surface area for excreta collection, involving swabs or multiple large FTA cards (30) that remain unable to efficiently sample all trapped mosquitoes. Additionally, preliminary experiments from the Price lab indicate that FTA reagent on the cards is toxic to mosquitoes and often results in morbidity and death before excretion can occur (Price, personal observation). This setup increases the work required to retrieve and process a sample, increases the potential for environmental contamination, and may miss a significant fraction of the excreta, since unlike with saliva collection, where the mosquito interacts directly with the card via feeding, excreta collection depends on randomly released droplets of exceedingly small volumes. A proposed solution that proved effective in the lab involves collecting and funneling the excreta into an Eppendorf tube using a paper cone treated with an over-the-counter hydrophobic waterproofing spray (31, 32). However, this approach may not be as feasible for long-term, semi-permanent field use, where the paper must withstand more intense environmental conditions than in the laboratory.

An additional limitation for most existing arbovirus surveillance methods, including FTA card and excreta-collecting techniques discussed above, is a reliance on qPCR for detection. This procedure requires species-specific PCR primers for pathogens chosen *a priori* by investigators (13). The rapidly changing vector-borne disease landscape is difficult to predict, as threats consistently spill over from zoonotic cycles, are introduced in unexpected places, and undergo genetic change (4, 29), which cannot be exposed using a responsive method of detection, such as qPCR. In these cases, a more comprehensive approach to molecular detection of vector-borne pathogen surveillance would greatly improve foresight and increase potential outbreak preparedness.

Next-generation sequencing (NGS) technology has made rapid advancements: becoming cheaper, faster, and achieving greater outputs with longer reads (31, 33), and is now feasible for pathogen discovery and surveillance (32). Such protocols have been incorporated into vector-enabled metagenomic approaches using mixed-species mosquito samples, enabling identification of a wide range of animal, plant, insect, and bacterial viruses (34). NGS has also been used to identify non-mosquito-borne viruses, such as Epstein-Barr and canine distemper, from blooded mosquitoes collected inside homes (35) and in metabarcoding approaches recovering mosquito species ID and infection statuses from large mixed pools (36). NGS performed on excreta from experimentally infected mosquitoes and field samples determined that the former provided sufficient template for sequencing and assembly of near-full-length genomes, while the latter could detect arbovirus sequences in samples with C_T_ values ≤ 28.5, possibly due to lower sensitivity in NGS when compared to RT-qPCR (37).

Here, we develop and test a rigid, permanent superhydrophobic excreta collection funnel for lab use. Populations of field-caught *Culex* mosquitoes were suspended over our device to efficiently aggregate and collect all excreta into an attached Eppendorf tube. Through shotgun metagenome sequencing of this template, we uncovered a rich and diverse RNA virome, with additional analyses to confirm the mosquito species from which the samples were collected and the trypanosomatid parasites they harbored. We envision methods and devices such as these becoming widely used in future surveillance practices, particularly in remote areas where repeated collections are difficult. Through our results, we emphasize how this kind of monitoring can help detect emerging and/or unknown pathogens of One Health significance, expand our understanding of mosquito virome biogeography, and identify as-yet undescribed viruses.

## MATERIALS AND METHODS

**Fig 1:**
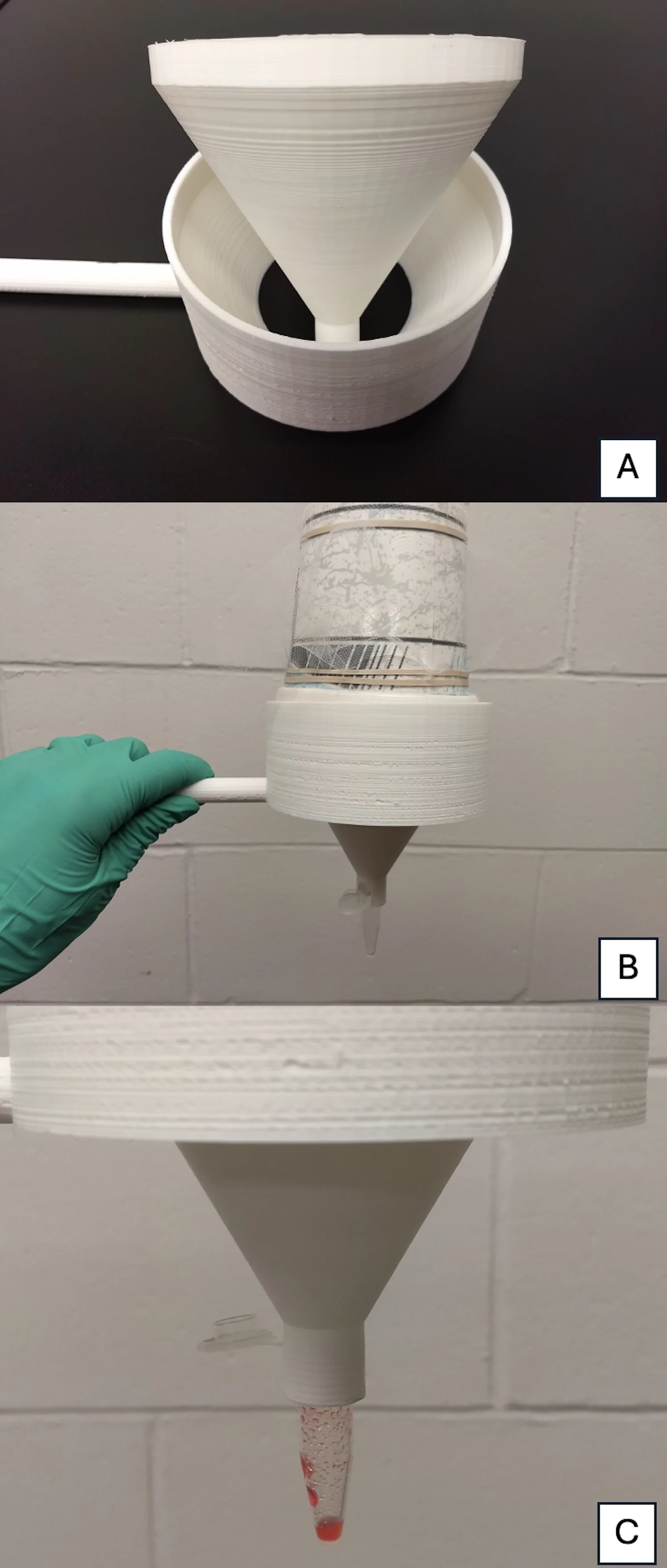
Excreta collecting device. (**A**) PLA-printed funnel with ring-stand collar adapter. (**B**) Funnel with inverted, screened cardboard soup container containing 50 *Culex* mosquitoes. (**C**) Collected excreta in attached microcentrifuge tube. Red coloration is due to red food dye added to sucrose solution upon which mosquitoes fed.

A funnel-type collection device was designed and fabricated using Form 3 Clear Resin V4 via 3D printing technology (Formlabs Inc., Somerville, MA; Fig. 1) with an overall diameter of 4.1 inches, a length of 4.0 inches, and a conical taper angle of 60 degrees, providing a compact yet efficient design. The funnel spout was outfitted with a collar, which functions to firmly attach a standard 1.5 mL microcentrifuge tube for excreta collection. After fabrication of the funnel via 3D printing, the interior surface was refined using 320 and 1,000 grit sandpapers for a smooth finish. The interior of the funnel was then coated with superhydrophobic spray (HIREC 1450-NF, NTT Advanced Technology Corp., Tokyo, Japan) twice and cured in an oven at 60°C for 1 h.

Two pools of *Culex pipiens* complex mosquitoes were collected from an urban yard in New Brunswick, NJ (40°28′45.8″N 74°26′36.7″W), on 12 September 2021 and 21 September 2022, respectively, using a Reiter-Cummings gravid trap baited with hay infusion. Fifty mosquitoes were removed from the trap, visually identified as *Culex pipiens* (including forms pipiens and molestus), and aspirated into cardboard soup containers with both the top and bottom surfaces cut out and replaced with UV-sterilized bridal’s veil. The container was inverted (bottom side up), placed in the funnel, and a sterile 1.5 mL conical Eppendorf tube was attached to the base of the funnel (Fig. 1B and C). A 1-inch section of cotton dental dam was soaked in autoclaved 10% sucrose solution with several drops of red food coloring and placed atop the chamber on the bridal’s veil, and the funnel + container were placed in an alcohol-sterilized dead air box at room temperature for 18 h. After feeding on the sucrose solution overnight, the Eppendorf tube was removed from the funnel and briefly spun down in the centrifuge; approximately 45–55 µL of red-dyed excreta was visible in each tube.

The volume of excreta was brought to 200 uL using sterile PBS and passed through an Omega Homogenizer column (Omega Bio-tek, Norcross, Georgia). Total nucleic acids were extracted from the lysate using the QIAamp MinElute Virus Kit (Qiagen, Venlo, the Netherlands) per manufacturer protocol with final elution in 20 uL of buffer AVE. A volume of 10 uL of the extraction product was treated with Turbo DNase (Thermo Fisher Scientific, Waltham, MA) per manufacturer protocol and converted to enriched cDNA using the Sigma WTA whole-transcriptome amplification kit (Sigma-Aldrich, Burlington, MA). An Illumina next-generation shotgun sequencing library was prepared with the Illumina DNA Sample Prep Kit (Illumina, Inc., San Diego, CA) per manufacturer protocol and quantified on a Qubit fluorometer. An additional DNA library was created from the 2020 sample by treating a 10 uL aliquot of the extraction product with RNase A (Ambion) prior to library preparation with the Illumina DNA Sample Prep kit.

Raw Illumina sequencing reads were quality- and adapter-trimmed using BBduk of the BBtools package (https://archive.jgi.doe.gov/data-and-tools/software-tools/bbtools/) with parameters “minlen = 50 qtrim = rl trimq = 15 ktrim = r k = 12 mink = 6.” To speciate the mosquitoes using excreta, we first overlapped the trimmed paired reads into a single amplicon (where possible) with BBmerge of the BBtools package (parameters “forcetrimright2 = 20”) and performed a BLASTn query (*e*-val = 1 × 10^−10^) of the merged amplicon to all Culicidae extracted from a local copy of the public BOLD database (38); database dated 04 October 2024. The top hits were saved and those with a local alignment spanning at least 300 nucleotides were retained for analysis.

To identify viral sequences present in our results, we assembled the trimmed reads with SPAdes 3.15.5 (39) using parameters “--meta -k 33, 53, 73, 93, 113, 127.” The resultant scaffolds were queried against a database consisting of the NCBI “nt” nucleotide database (downloaded January 2025) using BLASTn (40) with an *e*-val = 1 × 10^−5^. To exclude potential endogenous viral elements that can be pervasive in vector arthropod genomes (41, 42), we added all available *Culex* genome sequences and those of *Aedes albopictus*, *Ae. aegypti*, *Ae. notoscriptus*, *Ae. koreicus*, and *Ae. japonicus* to the database (Table S1). A second homology search against the NCBI “nr” protein database was conducted using DIAMOND (43) with parameters “--evalue 1e-5 --very-sensitive.” Scaffolds with top hits to viruses via both the BLASTn and DIAMOND queries were retained for discussion here. If either assembly contained multiple scaffolds that together appeared to span the full length of a single virus based on their top hits, the raw reads for this library were mapped to the BLAST reference using BBmap within Geneious Prime v. 2025.2 (parameters: vslow = t [“Highest sensitivity/slowest”], “Map multiple best matches to none”) and the read mapping was manually examined for contiguous coverage and identity (i.e., lack of segregating polymorphisms and misalignment or quasi-species). A majority-rule consensus contig was then exported for deposition and further study below. We retain for discussion here viral scaffolds that are likely to represent complete or near-complete genomes (or genome segments) in either funnel assembly based on length relative to their respective BLASTn top hits and/or the creation of a contiguous viral contig via short-read mapping and consensus extraction. Consensus viral genome sequences deposited to GenBank (Table 1) consist of those from whichever sample (funnel 1 or 2, if recovered in both) yielded the longest contiguous sequence. Unless otherwise noted, genomes recovered from both funnel samples, if present as such, were not directly compared to each other in tests of sequence conservation or evolution. Confirmatory West Nile virus TaqMan qPCR assays were performed using the RNA extractions of both pools as in Lanciotti et al. (44) with both the WNENV (envelope) and WN3′NC (3′ non-coding) primer/probe sets on an ABI QuantStudio 5 quantitative PCR instrument.

**TABLE 1:** Viruses recovered from mosquito excreta, assigned NCBI accession numbers, novelty, sample representation, and top-hit protein homology scores to the NCBI “nr” database

Class	Virus	Accession	Length	Novel?	Funnel 1/2	NCBI top hit	NCBI organism	% sim	aln len	*e*-val
3	Ahus virus	PX597326	7,728		1+2	MK440642.1	Ahus virus isolate OTU52	97.787	7,727	0
3	Black Hawk Lake virus	PX597327	1,712		1+2	OM743939.1	Black Hawk Lake virus isolate BHLV-2020	99.883	1,712	0
3	Unidentified Partiti-like virus	PX917205	1,571	Yes	1+2	MN661055.1	Atrato Partiti-like virus 1 strain Mati 1755-173	66.026	624	1.70^−30^
3	Unidentified Partiti-like virus	PX917206	1,720	Yes	1+2	NC_033314.1	Hubei tetragnatha maxillosa virus 8 strain arthropodmix13417	70.69	174	2.78^−09^
4	Culex mosquito virus 1	PX597328	1,856		1+2	MH188026.1	Culex mosquito virus 1 strain CMosV1/Santa Cruz	99.084	1,856	0
4	West Nile virus	PX597329	10,974		2	MF175875.1	West Nile virus isolate AVA1506113	99.435	10,979	0
4	Marma virus segment RNA1	PX597330	3,155		1+2	MW434881.1	Marma virus isolate CMS001_044_ALCO	99.334	3,154	0
4	Marma virus segment RNA2	PX597331	1,647		1	MW434843.1	Marma virus isolate CMS002_051a_PLCR	99.817	1,643	0
4	Houston virus	PX613661	20,139		1	MH188023.1	Houston virus strain HV/Solano	99.761	20,097	0
4	Culex Iflavi-like virus 4	PX613662	9,826		1	NC_040574.1	Culex Iflavi-like virus 4 strain CIVL/Kern	98.142	9,686	0
4	Culex pipiens-associated Tunisia virus	PX613663	11,023		1+2	MW434969.1	Culex pipiens-associated Tunisia virus isolate CMS002_047h_WVAL	99.194	10,916	0
4	Culex-associated Tombus-like virus	PX613664	2,649		1+2	NC_040575.1	Culex-associated Tombus-like virus strain CaTomVL/Santa Cruz	99.315	2,628	0
4	Unidentified picorna-like virus	PX613665	9,569	Yes	1+2	MN661035.1	Atrato picorna-like virus 1 strain An 1771-1	87.731	9,561	0
4	Negev virus	PX625216	9,508		1	JQ675605.1	Negev virus strain EO-329	98.433	9,507	0
4	Cordoba virus	PX625217	7,486		1	KX518771.1	Cordoba virus strain GMC30	99.292	7,485	0
5	Hedwig virus segment L partial	PX640301	6,543		2	MK440651.1	Asum virus isolate OTU76	97.003	1,935	0
5	Hedwig virus segment M partial	PX640302	4,169		2	OZ335791.1	Hedwig virus strain UKMosq_110b	96.905	4,168	0
5	Hedwig virus segment S partial	PX640303	1,170		2	OZ335792.1	Hedwig virus strain UKMosq_110b	95.086	1,160	0
5	Unidentified leishbuvirus segment L	PX640304	5,857	Yes	2	MG967334.1	Blechmonas luni leishbunyavirus 1 strain OSU1	69.737	304	6.06^−24^
5	Unidentified leishbuvirus segment S partial	PX640305	481	Yes	2	OR208640.1	Crithidia bombi leishbuvirus 1 isolate 12-450	73.451	113	1.09^−07^
5	Hapavirus flanders	PX661632	12,661		2	MH664048.1	Hapavirus flanders isolate CT-460	98.247	12,662	0
5	Culex phasma-like virus segment L	PX666094	5,904		2	MN661018.1	Culex phasma-like virus strain Cx 1773-14	98.689	2,975	0
5	Culex phasma-like virus segment M	PX666095	1,907		2	MF176354.1	Culex phasma-like virus strain mosWSgb51997	98.898	1,906	0
5	Culex phasma-like virus segment S	PX666096	1,987		2	MN661019.1	Culex phasma-like virus strain Cx 1773-14	99.295	1,986	0
5	Wuhan mosquito virus 6 segment 1	PX675826	2,438		1	MW434416.1	MAG: Wuhan Mosquito Virus 6 isolate CMS001_028_ALCO	99.179	2,437	0
5	Wuhan mosquito virus 6 segment 2	PX675827	2,433		1	MW434433.1	MAG: Wuhan Mosquito Virus 6 isolate CMS002_029c_WVAL	98.767	2,433	0
5	Wuhan mosquito virus 6 segment 3	PX675828	2,219		1	MW434407.1	MAG: Wuhan Mosquito Virus 6 isolate CMS001_034_ALCO	98.963	2,219	0
5	Wuhan mosquito virus 6 segment 4	PX675829	1,870		1	MW434385.1	MAG: Wuhan Mosquito Virus 6 isolate CMS002_056a_PLCR	99.031	1,858	0
5	Wuhan mosquito virus 6 segment 5	PX675830	1,500		1	MW434443.1	MAG: Wuhan Mosquito Virus 6 isolate CMS002_056a_PLCR	98.664	1,497	0
5	Wuhan mosquito virus 6 segment 6	PX675831	853		1	MW434448.1	MAG: Wuhan Mosquito Virus 6 isolate CMS001_032_ALCO	99.648	853	0
5	Wuhan mosquito virus 6 segment 7	PX675832	644		1	MW434467.1	MAG: Wuhan Mosquito Virus 6 isolate CMS002_029e_WVAL	99.068	644	0
5	Wuhan mosquito virus 6 segment 8	PX675833	587		1	MW434479.1	MAG: Wuhan Mosquito Virus 6 isolate CMS001_043_ALCO	98.978	587	0
N/A	Biggievirus Mos11	PX675834	9,160		1+2	MF281708.1	Biggievirus Mos11 strain 258719/2008	99.692	9,082	0

To screen for trypanosomatid parasites, we performed a BLASTn query (*e*-val = 1 × 10^−10^) of the merged amplicons (generated during our mosquito speciation step) to the SILVA 18S small subunit ribosomal RNA database v.138.2 (45). Amplicons with top hits of at least 98.0% nucleotide similarity spanning a local alignment length of at least 300 nucleotides were retained. The top query (i.e., merged read pair) for each SILVA reference that generated a hit was extracted, reduced to a non-redundant set (*n* = 35 amplicons), and aligned together using the pre-aligned trypanosomatid 18S SSU sequences (*n* = 296) in the SILVA database as a template, using MAFFT v.7.5 (46) with parameters = “--add-fragments --adjustdirectionaccurately.” A maximum-likelihood phylogeny was constructed from this alignment using IQTREE v. 3.0.1 (47) with parameters = “-bb 2000 -nm 2000 -m TEST” using automatic model selection and 2,000 rapid bootstrap approximations.

## RESULTS AND DISCUSSION

We generated 15.42 M paired-end DNA and 16.54 M paired-end RNA reads of length 250 bp from the 2021 (Funnel 1) sample and 42.0 M paired-end RNA reads of length 300 bp from the 2022 (Funnel 2) sample. Assembly of each library using SPAdes resulted in 23,159 scaffolds totaling 13.54 Mbp (N50 = 561 bp) for the Funnel 1 RNA library, 37,343 scaffolds totaling 54.13 Mbps (N50 = 2.84 Kbp) for the Funnel 1 DNA library, and 13,194 scaffolds totaling 11.84 Mbp (N50 = 1.02 Kbp) for the Funnel 2 RNA library. Below, we discuss the likely taxonomic provenances of sequences related to the host insect, its viruses, and its trypanosomes, highlighting ways in which they suggest our surveillance approaches could be applied. All viral sequence data discussed below have been deposited to the NCBI GenBank database with accessions and homology output listed in Table 1 and abundance in Table 2; full homology search output in Table S2. We did not identify viruses other than bacteriophage in the DNA library. As we selected for discussion here the viral scaffolds reflective of complete or near-complete segments and/or genomes based on the homology search, it is very likely that we have under-sampled the true diversity of viruses in our samples, as could be accomplished by, for example, phylogenetic analyses of all assembled fragments with top hits to viruses. Further to this point, the general efficacy of excreta and shotgun sequencing for broad virus discovery itself remains unproven under laboratory controlled, empirical studies that, for example, quantify limits of detection for viral recovery from experimentally infected mosquitoes with known viruses at varying viremia. It should be noted that we cannot infer the manner of infection or tissue tropisms of any viruses discussed herein that currently lack such data, as both disseminated and transient viral infections, in addition to viruses that have simply passed through the gut undisseminated, could also be present in excreta.

**TABLE 2:** Total number of RNA reads mapped, and percent of the total library for each of Funnel 1 (F1) and Funnel 2 (F2), to each viral scaffold discussed herein

Virus	F1 reads	%	F2 reads	%
Ahus virus	4,270	0.01	5,396	0.03
Biggievirus Mos11	259,860	0.80	21,184	0.10
Black Hawk Lake virus	4,826	0.01	4,592	0.02
Cordoba virus	1,362	0.00	0	0.00
Culex-associated Tombus-like virus	708,300	2.17	653,027	3.20
Culex Iflavi-like virus 4	28,988	0.09	2	0.00
Culex mosquito virus 1	2,266,393	6.96	1,154,688	5.66
Culex phasma-like virus segment L	10	0.00	134	0.00
Culex phasma-like virus segment M	270	0.00	1,578	0.01
Culex phasma-like virus segment S	70	0.00	170	0.00
Culex pipiens-associated Tunisia virus	1,184	0.00	4,802	0.02
Hapavirus flanders	0	0.00	1436	0.01
Hedwig virus segment L partial	0	0.00	212	0.00
Hedwig virus segment M partial	0	0.00	586	0.00
Hedwig virus segment S partial	0	0.00	130	0.00
Houston virus	508,731	1.56	4	0.00
Marma virus segment RNA1	4,763	0.01	2,042	0.01
Marma virus segment RNA2	2,762	0.01	334	0.00
Negev virus	35,397	0.11	2	0.00
Unidentified picoRNA-like virus	1,439,901	4.42	8,764	0.04
Unidentified leishbuvirus segment L	4	0.00	1,026	0.01
Unidentified leishbuvirus segment S partial	0	0.00	186	0.00
West Nile virus	28	0.00	42,782	0.21
Wuhan mosquito virus 6 segment 1	2,434	0.01	8	0.00
Wuhan mosquito virus 6 segment 2	3,743	0.01	6	0.00
Wuhan mosquito virus 6 segment 3	2,843	0.01	20	0.00
Wuhan mosquito virus 6 segment 4	4,133	0.01	4	0.00
Wuhan mosquito virus 6 segment 5	326	0.00	0	0.00
Wuhan mosquito virus 6 segment 6	1,242	0.00	6	0.00
Wuhan mosquito virus 6 segment 7	1,089	0.00	0	0.00
Wuhan mosquito virus 6 segment 8	97	0.00	0	0.00

### COI screening for mosquito species identity

Screening of the overlapped reads using BLASTn against the BOLD database (comprising the 5′ and 3′ barcoding loci) generated hits for 13,959 reads from all three libraries (Table S3), for which 13,788 (98.8%) were to *Culex pipiens* or *Culex quinquefasciatus* (10,361 to *Culex pipiens,* 3,427 to *Culex quinquefasciatus*); all remaining hits were to a mix of *Culex* species (*Cx. declarator*, *Cx. brami*, *Cx. chidesteri*, *Cx. mollis*, *Cx. salinarius*, *Cx. bilineatus*). Of the 8,361 hits to the 3′ COI locus, 8,337 (99.7%) were to *Cx. pipiens,* whereas of the 5,598 hits to the 5′ locus, 3,426 (61.2%) were to *Cx. quinquefasciatus,* and 2,019 (36.1%) were to *Cx. pipiens*. Manual examination of the full BLAST output for the hits to *Cx. quinquefasciatus* indicated that an equivalent top-scoring hit to *Cx. pipiens* existed as well. We thus suspect representation of *Cx. quinquefasciatus* is a BLAST artifact, especially since this species is not reported from our collection site in New Jersey (northeastern United States). A greater taxonomic diversity was witnessed in top hits to the 5′ locus; a manual BLASTn of the hits to non-native species *Culex brami* (35 hits), *Culex chidesteri* (11 hits), and *Culex declarator* (21 hits) against the NCBI “nt” database indicated that these amplicons, in fact, had top hits with >99% identity to *Culex restuans*, while those to *Culex mollis* aligned with >99% identity to *Culex salinarius*, exclusively. Overall, these results affirm that sequence data from excreta gathered through this method can be used to help confirm host species identity (as in reference 36); however, artifacts may currently persist as a factor of database compositional biases and/or curation and identification errors that require corroboration from different data sources. Further experimentation as to the limits of detection using excreta from pools of known species compositions will be necessary to assess the utility of such data when gleaned from sites with a broad mosquito species diversity.

### Detecting known or potential arboviruses in mosquito excreta samples

One of the major goals of this type of surveillance is to detect the circulation of arboviruses in important vector populations. We demonstrate the potential utility of our excreta collection method for this purpose in identifying a complete, high-coverage (>900×) West Nile virus (Amarillovirales, Flaviviridae) genome from our funnel 2 assembly. Not only were we able to detect this virus in our results, but using the polymorphisms identified within the short-read mapping, we were able to identify specific amino acid substitutions associated with WNV genotype NY07 (T1195I, L1238F, S1839T, S2287I), a variant identified in New York from pooled, homogenized female mosquitoes (48). We thus suggest that, given sufficient representation in the sample, this method can achieve a level of resolution high enough to potentially enable strain-level detection of important arboviruses. Our TaqMan qPCR assay confirmed that the Funnel 2 sample was positive for WNV (WNENV and WN3′NC Ct values = 25.19 and 24.51, respectively), while the Funnel 1 sample was negative.

In addition to well-characterized pathogens like West Nile virus, this type of sampling can also help us detect more obscure arboviruses that may be of public health interest with regard to potential spillover into human or livestock populations. For example, in our results, a group of six scaffolds from the funnel 2 assembly had top hits to Hedwig and/or Asum virus, both representatives of *Gryffinivirus hedwigae* in the novel genus *Griffinivirus* (Elliovirales, Peribunyaviridae) (49), that have thus far only been detected in Europe (50–52). This genus was first described from snowy owls (*Bubo scandiacus*) co-infected with West Nile virus and autopsied in Germany, suggesting that at least some members may be capable of infecting vertebrates.

Of the gryffiniviruses*,* only Udune virus (*G. alamedaense*) has been reported in the United States (49, 53); a BLASTn search revealed that its genome shared relatively low BLASTn nucleotide identity with our scaffolds (ca. 71.0%–78.8%, not shown). By contrast, the S (1.07 Kbp, 26.8× cov.), M (4.17 Kbp, 32.9× cov.), and L segments (1.90 Kbp, 17.0× cov.) reported here shared a comparatively high nucleotide sequence identity with the references for Hedwig and Asum viruses (95.0% and 97.0%, respectively). Both the S and M segments spanned 86.4% and 91.7% of the lengths of these references, although the L segment fragment corresponded to only 26.6% of its corresponding NCBI top hit. However, this improved to 94.7%, 94.0%, and 81.6%, respectively, after we generated consensus contigs via short-read mapping to NCBI references OV055471, OV055470, and MK440651 (segments S, M, and L [50]). Thus, we suggest that the viruses identified here are unlikely to be related to be Udune virus and instead represent what is, to our knowledge, the first report of Hedwig-like viruses from the Americas.

### Expanding geographic data to contextualize the global mosquito virome

As demonstrated above, the information obtained using analyses such that this can be used to enhance our understanding of the biogeography of mosquito-associated viruses, even beyond those likely to produce disease in vertebrates. For example, the most abundant virus by depth of coverage in our analysis was Culex mosquito virus 1 strain Santa Cruz (Nodamuvirales, Nodaviridae), which has, to our knowledge, only previously been recorded from *Culex* spp. in California (54) and a pool of *Cx. pipiens* in Germany (51). Assemblies from both funnels contained scaffolds with full- or near-full-length viral genomes with amino acid and nucleotide identities of >99% and average read coverages of >140,000×, suggesting that it was not only present in our samples but highly abundant. Additionally, many shorter scaffolds with top hits to this virus were also present in both funnels, likely due to over-assembly resulting from such deep coverage. To more accurately visualize the contiguity of our data with respect to the reference, we mapped the raw reads to the Culex mosquito virus 1 strain CMosV1/Santa Cruz genome (NCBI accession MH188026). Both mappings confirmed full-length reference coverage at >244,000× and >175,000× in funnels 1 and 2, respectively.

In some cases, our data represent the first additional records of some mosquito-associated viruses since their initial description. These include sequences with high coverage (480×) and high percent nucleotide identity (nearly 100%) to a Black Hawk Lake Virus (BHLV, unclassified) reference first recorded and described as an RdRp-encoding scaffold in a metagenomic sample from Iowa, USA (55), and to an Ahus virus genome (Ghabrivirales, Orthototiviridae, 95× and 150× in each funnel, respectively, at 97.9% nucleotide identity) first described by Petterson et al. (50) from meta-transcriptomic sequencing of *Culex* mosquitoes in Sweden. The latter was suggested by the authors to be an environmental contaminant due to low coverage and poor representation in their overall sampling scheme, as Totiviridae and related families are predominantly associated with fungi and protozoa. However, in more recent studies, members of these families have also been reported from insects and other arthropods (56), and in our results, high coverage and presence in both funnels support a potentially genuine association of Ahus virus with *Culex* mosquitoes.

Other viruses that we detected in our results are already known to be broadly distributed across global mosquito populations. For example, we identified scaffolds which corresponded to sequences from Marma virus (Tolivirales), which has been recorded in *Culex* from Europe, Mexico, and the United States (50, 53); *Culex-*associated Tombus-like virus (Tolivirales, Tombusviridae), which has been reported from California, the United Kingdom, and Australia (54, 57); Houston virus strain HV/Solano (Nidovirales, Mesoniviridae), which is part of a viral lineage that has been detected in the USA (Texas), Java, Indonesia, Vietnam, and Côte d’Ivoire (58); *Culex* Iflavi-like virus 4 (Picornavirales, Iflaviridae), which has been identified in samples spanning 12 countries and four continents (59), including from *Culex* in the USA (California) and Canada (54, 60); *Culex pipiens*-associated Tunisia virus (CpATC, unclassified), which has been reported from *Culex* in Tunisia, USA (California), and Brazil (53, 61), as well as *Anopheles epiroticus* from Vietnam (59, 62); and Wuhan mosquito virus 6 (Articulavirales, Orthomyxoviridae), which is so broadly distributed that it has been hypothesized to be one of the relatively few viruses belonging to a vertically transmitted “core” mosquito virome (63, 64). Our Marma virus assemblies bore >99.3% nucleotide identity to complete (funnel 1) or near-complete (funnel 2) segment RNA1 and >99% identity to segment RNA2 (in funnel 1), with the presence of the full viral genome in funnel 1 confirmed via short-read mapping. The *Culex*-associated Tombus-like virus scaffolds each had nucleotide identities of 99% or greater to the reference and an average coverage of 25,000–50,000×. By mapping the raw data from funnel 1 to the *Culex*-associated Tombus-like virus strain CaTomVL/Santa Cruz reference (NCBI accession NC_040575.1), we generated a consensus 2,649 bp contig with mean read coverage of 54,556× and 99.0% pairwise identity to the reference. For Houston virus, we identified a 20.2 Kbp scaffold matching the complete genome reference sequence at 99.75% nucleotide identity and >4,600× average coverage. A complete genome of *Culex* Iflavi-like virus 4 with a top BLASTn hit to strain CIVL/Kern (California) was represented as a single 9.82 Kbp scaffold (98.14% nucleotide identity, 524× average coverage). A 10.9 Kbp linear scaffold constituting the genome of *Culex pipiens*-associated Tunisia virus was identified at 107× average coverage and 99.2% nucleotide identity in the funnel 2 assembly, while read mapping to the NCBI reference genome confirmed full recovery of the genome in funnel 1 as well. Eight scaffolds homologous with each of the eight segments that comprise the complete genome of Wuhan Mosquito Virus 6 were identified in the funnel 1 assembly. Consensus scaffold lengths ranged from 91% to 100% of their respective NCBI references, with nucleotide sequence identities of 98.66% (segment 5) to 99.65% (segment 6). Six of the eight scaffolds had average sequence coverage of >220×. While five scaffolds within the funnel 2 assembly also had top hits to this virus, their lengths were much shorter and read coverage was substantially lower at <4×.

In addition to these globally prevalent virome members, we also document viruses with fairly consistent but (at the time of this writing) geographically restricted records. For example, three scaffolds were identified in the funnel 2 assembly that together spanned the entire length of the linear 13.04 Kbp genome of Flanders virus (FLAV; Mononegavirales, Rhabdoviridae), which had previously been detected from *Culiseta melanura* mosquitoes and avian spleens in Flanders, NY (65), and from *Culex tarsalis* in multiple US states and Canada (66). Mapping of our short-read data to the *Hapavirus flanders* isolate CD-460 genome (NCBI accession MH664048) indicated that we had a mean coverage of 27× spanning 97.0% of the reference, and the consensus 12.64 Kbp contiguous viral genome fragment we generated was 99.0% identical at the nucleotide level. These data also allowed us to place our record within one of two distinct, co-circulating viral lineages (67, 68): the U1 nucleotide CDS sequence had a top BLASTn hit (99.6% nucleotide identity, 100% query length) to Lineage A, Flanders virus strain CHC2302-05/GA/2005 isolated from *Culex quinquefasciatus* in Georgia, USA.

### Detecting novel mosquito viruses and current arbovirus control candidates

In some cases, we not only identified novel records of existing strains but also uncovered undescribed viruses in our *Culex* excreta. As there was a clear bifurcation in the degree of BLASTp amino acid identity of our viral scaffolds to those in the NCBI nr database (less than 88% or greater than 95%), we have designated those viruses with <88% amino acid identity as putatively novel herein. For example, we identified two scaffolds encoding segments of a partiti-like virus (Durnavirales, Partitiviridae) in each funnel, with a complete capsid protein-encoding segment assembled in both funnels 1 and 2 at high coverage (1,677× and 709×, respectively) yet low amino acid identity (54% in each) to Atrato partiti-like virus 1. A scaffold encoding an associated “hypothetical protein 2” with top hits to Atrato partiti-like virus 1 was identified only in funnel 2 with an average coverage of 2,835× and 53% amino acid identity. Typically, *Partitiviridae* genomes consist of two segments of monocistronic dsRNA, but partiti-like viruses described from *Mansonia titillans* mosquitoes in Colombia comprise four segments with two additional hypothetical proteins in addition to the standard capsid and RNA-directed polymerase (RdRP) genes (NCBI accessions MN661051–MN661056). Because of the very low amino acid homology between the capsid and hypothetical proteins reported here and existing GenBank references, we propose that the virus identified in our sample is putatively novel but may possess additional viral genome segments (in particular, encoding the RdRP) that we were unable to assemble; pending further sampling that suggests these two segments are linked, we have assigned them separate isolate identifiers for deposition.

Beyond this partiti-like virus, we also detected a putatively novel picorna-like virus in our samples. We found that both funnel assemblies contained scaffolds with top BLASTn hits to Atrato picorna-like virus 1 (Picornavirales; unassigned). The single 9.72 Kbp scaffold (207× average coverage) from the funnel 2 assembly encoded a complete polyprotein and exhibited 88.0% nucleotide identity over 99% of the complete reference genome, while the funnel 1 assembly contained two scaffolds (3.41 and 6.59 Kbp, average nt coverage of 17,853× and 29,604×) that together spanned the full length of the reference at 87.0% to 88.0% nucleotide identities. Short-read mapping to the picorna-like virus strain An1771-1 reference (NCBI accession MN661035) generated a consensus contig that maintained 86.5% nucleotide identity. While Atrato picorna-like virus 1 has not been reported from North America, a similar yet diverged virus, Zhejiang mosquito virus isolate Cx.erraticus17, was recovered from *Cx. erraticus* in Alabama (NCBI accession PQ963484.1). However, our scaffolds maintained a similarly low nucleic acid identity to this virus as well (87.0%). Given the low sequence conservation between the recovered viral genomes and any existing reference, we designate this virus as likely belonging to an as-yet undocumented picorna-like virus.

We detected highly divergent viral genomic data even within lineages with well-characterized members already documented in the results. For example, multiple scaffolds in both assemblies had top hits to each of the three segments comprising the Culex phasma-like virus (Elliovirales; Phasmaviridae) genome. While the BLASTn alignments for the funnel 2 scaffolds averaged >99% nucleotide sequence identity, those from funnel 1 were much lower scoring at 51%–87%. Mapping the funnel 2 short-read data to the genome of Culex phasma-like virus strain Cx 1773-14 (NCBI accessions MN661018–MN661020) confirmed complete and contiguous read coverage over the S (17× cov., 98.3% nt identity), M (190× cov., 99.0% nt identity), and L (5× cov., 99.5% nt identity) segments. The majority of reads from funnel 1, however, had very low homology to the reference to the extent that they remained unaligned, and we could not generate a consensus nor confirm a taxonomic provenance for that virus.

Surveilling novel members of the mosquito virome may yield candidates for interrupting transmission of arboviruses to vertebrates. For example, both funnel assemblies contained many scaffolds of greater than 1 Kbp with top hits to the Culex flavivirus (CxFV; Amarillovirales, Flaviviridae) genome and nucleotide identities of >95%. This is a broadly distributed member of a viral complex known as the insect-specific flaviviruses, which includes cell-fusing agent virus and other related viruses that have been shown to co-circulate and interfere with mosquitoes’ abilities to vector dengue, Zika, and West Nile viruses (68–71). Mapping of our raw Illumina data to the Culex flavivirus strain CFV/San Francisco genome (NCBI accession MH188006) yielded a mean coverage of 560× and 165× for each of the funnel 1 and 2 libraries, with consensus pairwise identities of 94.6% and 97.4% that spanned 99.7% and 99.5% of the reference, respectively. A high degree of polymorphism was evident in the mapping, with 272 sites in the funnel 1 mapping and 240 sites in the tunnel 2 mapping exhibiting SNP frequencies between 25% and 75% in the raw reads. We suspect that a diversity of quasi-species is present in our samples, leading to fragmentation of the viral genome assembly; for this reason, we did not generate a consensus contig, as the likelihood of a chimeric consensus was high. In funnel one, we also found scaffolds corresponding to Negev virus strain EO-329 and Cordoba virus (both unclassified at the level of order and family). The lineage to which these viruses belong comprises some of the most abundant insect-specific viral strains in *Culex* and *Aedes* mosquitoes, which have been evaluated for their effect on the replication of *Alphavirus*, Chikungunya virus, and eastern equine encephalitis virus in mosquito cells (72, 73). Each of the two scaffolds identified as Negev virus strain EO-329 in our results had >600× average coverage and 98.4% nucleotide identity. There were multiple scaffolds with top hits to Cordoba virus, and short-read mapping spanned 98.5% of the reference genome (Cordoba virus strain GMC30; NCBI accession KX518771) with average coverage of 31.6× and nucleotide identity of 99.3%.

Even where the virus in question is not necessarily a candidate for limiting arbovirus transmission, these metagenomic surveys advance biomedical science by providing new candidates for other kinds of functional applications. For example, we recovered one scaffold from funnel 1 and a pair of scaffolds in funnel 2 that spanned the entire 9.22 Kbp genome of Biggievirus Mos11 (unassigned) with a consensus genome recovered at 5,304× and 561×, respectively, at 99.70% nucleotide identity (98.0% query length) with Biggievirus Mos 11 strain 258719/2008 isolated from Italy. This virus has recently emerged as a tool for biotechnology research, as it encodes a novel phage-like hypercompact CRISPR-Cas type V-J nuclease designated CasΦ or Cas12j (74), and has been explored for genome-editing applications due to its small size and ease of cellular delivery (75).

### Detecting presence and virome of mosquito-associated microbial eukaryotes

**Fig 2:**
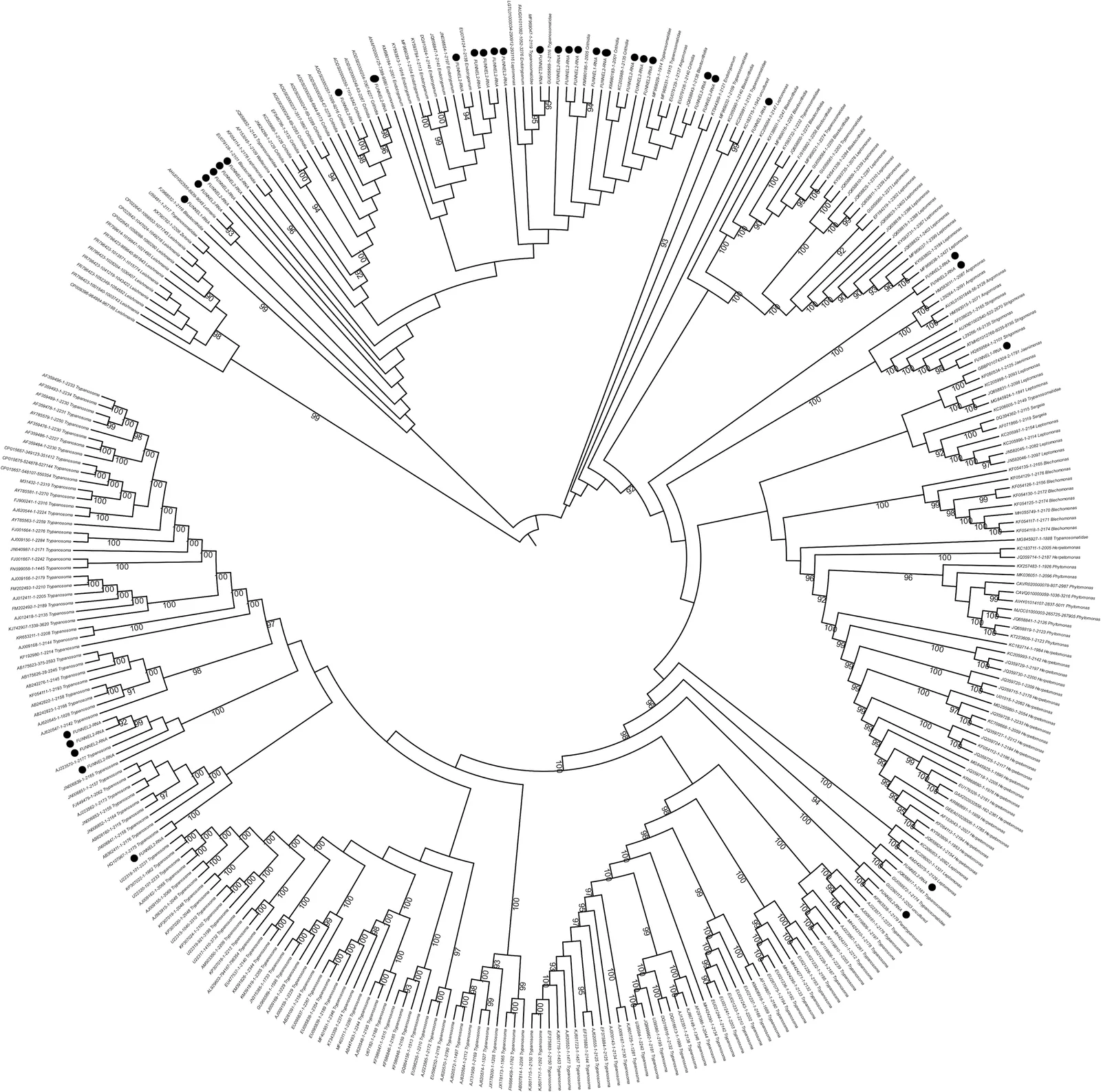
Trypanosome 18S SSU maximum likelihood phylogeny. Radial cladogram (branch lengths off) comprising all SILVA 138.2 trypanosomatid taxa and top-hit overlapped read pairs. The tree is manually rooted on Leishmania. Bootstrap supports >90 are shown. Linear tree with full taxon labels provided in Fig. S1.

In addition to viruses, we also surveyed our raw reads for trypanosomatids via 18S small subunit ribosomal RNA screening. BLASTn homology identified 92,963 merged reads as having >98.0% nucleotide identity over >300 bp of read length to 35 unique Trypanosomatida SSU references spanning multiple genera in the SILVA 138.2 database (Table 3). A phylogenetic analysis that comprised all 296 trypanosome references in the database and the non-redundant 35 single highest-scoring amplicons via BLASTn for all SILVA references demonstrates that multiple representatives of diverse trypanosome lineages are present in our samples (Fig. 2; Fig. S1). Phylogenetic analyses of trypanosome taxa commonly recover highly paraphyletic and polyphyletic assemblages of currently recognized genera (76, 77), and thus this analysis is not intended to recapitulate the trypanosome tree of life *per se*, but rather to illustrate the evolutionary diversity of 18S SSU amplicons identified in our samples on this backbone. The most abundant taxon by far, with 83,511 amplicon hits (22,894 from funnel 1 and 60,617 from funnel 2), was *Paratrypanosoma confusum* (avg 99.22% nt identity, 100% query length). First isolated from *Culex* mosquitoes in Prague, this species is the type species for genus *Paratrypanosoma* with a phylogenetic standing between free-living bodonids and the parasitic trypanosomatids (78). When manually queried against the NCBI ‘nt’ database (not shown), these amplicons returned top hits to *Paratrypanosoma* sp. isolate M2031 (79); 99.82% nt identity, 100% query length), isolated from *Culex* mosquitoes in Leningrad, Russia. Following *Paratrypanosoma confusum*, the second-most abundant protist was *Leptomonas pyrrhocoris* (2,526 amplicon hits; avg 99.20% nt identity, 100% query length), although this was present only in funnel 2. This is a monoxenous parasite of the hemipteran *Pyrrhocoris apterus* (80). Remaining top hits were to taxa with less than 500 total hits within the data. Given that we selected only the top-scoring BLASTn hit and reduced all hits to a non-redundant set of amplicons, it is likely that our analysis underestimates the true phylogenetic diversity of trypanosomes in our samples.

**TABLE 3:** Merged amplicon hits for both RNA libraries (BLASTn) to SILVA 138.2 SSU rRNA database trypanosomatid taxa^*a*^

Count	SILVA accession	Genus	Species
83,511	KF963538.1.2179	*Paratrypanosoma*	*Paratrypanosoma confusum*
2,526	LGTL01000034.290912.293116	*Leptomonas*	*Leptomonas pyrrhocoris*
377	AJ223570.1.2177	*Trypanosoma*	*Trypanosoma* sp*. N335*
339	HQ107967.1.2175	*Trypanosoma*	*Trypanosoma culicavium*
335	KM980184.1.2001	*Endotrypanum*	*Crithidia bombi*
214	KM980183.1.2001	*Crithidia*	*Crithidia mellificae*
207	DQ910924.1.2140	*Endotrypanum*	*Leptomonas neopamerae*
189	JN006839.1.2165	*Trypanosoma*	*Trypanosoma* sp*. LZ-2011*
176	KY593813.1.1916	*Endotrypanum*	*Trypanosomatidae* sp*. PNG136*
120	EU079129.1.2137	*Angomonas*	*Angomonas deanei*
102	KM980186.1.2005	*Crithidia*	*Crithidia expoeki*
62	ATMH01012768.6035.8195	*Strigomonas*	*Strigomonas culicis*
61	EU079126.1.2140	*Crithidia*	*Crithidia abscondita*
35	AODS02000237.3517.5697	*Crithidia*	*Crithidia fasciculata*
34	KF054114.1.2178	*Leptomonas*	*Leptomonas tenua*
30	MF969029.1.1914	*Unknown*	*Trypanosomatidae* sp*. PNG96f*
23	AF153045.1.2109	*Wallaceina*	*Wallaceina brevicula*
14	EU079128.1.2101	*Blastocrithidia*	*Blastocrithidia miridarum*
11	EU079124.1.2138	*Endotrypanum*	*Leptomonas cf. podlipaevi*
8	MF969031.1.1915	*Unknown*	*Trypanosomatidae* sp*. PNG98f*
7	EF546786.1.2132	*Crithidia*	*Leptomonas tarcoles*
6	AODS02000256.6944.9173	*Crithidia*	*Crithidia fasciculata*
3	AODS02000248.63.2267	*Crithidia*	*Crithidia fasciculata*
2	KC205990.1.2140	*Blastocrithidia*	*Leptomonas moramango*
2	GU059569.1.2116	*Unknown*	*Trypanosomatidae* sp*. Ch10*
2	CAVQ010000059.1036.3216	*Phytomonas*	*Phytomonas* sp*. isolate EM1*
2	AHIJ01002555.6839.9045	*Lotmaria*	*Lotmaria passim*
1	KR653211.1.2208	*Trypanosoma*	*Trypanosoma wauwau*
1	KF054129.1.2176	*Blechomonas*	*Blechomonas pulexsimulantis*
1	KC205989.1.2126	*Crithidia*	*Crithidia otongatchiensis*
1	KC205988.1.2135	*Crithidia*	*Crithidia pragensis*
1	JQ658843.1.2136	*Blastocrithidia*	*Trypanosomatidae* sp*. G67*
1	JQ359714.1.2187	*Herpetomonas*	*Lafontella mariadeanei*
1	DQ016616.1.2132	*Trypanosoma*	*Trypanosoma murmanensis*

^*a*^ Accessions are of the format “{accession}.{start coordinate}.{end coordinate}”.

Beyond the trypanosomes themselves, we identified two scaffolds in the funnel 2 assembly with weak protein homology to Duke bunyavirus (Hareavirales, Leishbuviridae), which is part of a family of viruses infecting trypanosomatid protozoan parasites (81). The scaffolds represent a full-length RNA-dependent RNA polymerase gene (5.86 Kbp, 40.54× cov.) and a partial nucleocapsid (481 bp, 80.78× cov), respectively, at very low amino acid identities of 39.5% and 55.5%. While the RdRp DIAMOND protein homology spanned 1,960 amino acids, neither scaffold had a high-scoring BLASTn hit (the larger scaffold had a 304 bp HSP to Blechmonas luni leishbunyavirus 1, while the nucleocapsid-encoding scaffold returned no BLASTn hits), indicating the nucleotide homology had dropped below the threshold of BLASTn identification. Although similar viruses have been identified in metatranscriptomic surveys of insects, spiders, and other invertebrates (82) in the past, suggesting the presence of infected protozoans in these hosts, the viral segment assembled here maintained very low amino acid identity to DuBV. Thus, although the plethora of trypanosome 18S SSU rRNA lineages in our data set suggests that more traditional prospective hosts are present, we cannot eliminate the possibility that a diverged, independent lineage of such viruses may infect insects directly.

### Conclusion

Our results from metaviromic sequencing of wild-caught *Culex* mosquito excreta using the device constructed here illustrate the potential utility of this protocol for detecting pathogenic arboviruses, elucidating viral biogeography, and enumerating and discovering mosquito viruses and other microbial constituents. We demonstrate that COI screening reveals sequences consistent with the identity of the mosquito species in our sample. We assemble a multitude of full-length (or near full-length) viral genomes and/or segments, many belonging to species that have never before been detected in the eastern United States—i.e., outside of California, where several mosquito metaviromic studies have already been conducted (53, 54), or even in North America (e.g., Hedwig virus). We describe putatively novel partiti-like and picorna-like viruses, as well as a possible novel leishbuvirus. Lastly, we highlight a broad diversity of trypanosomatid parasites, even from within a relatively small sampling effort at the same collection site.

While the trapped mosquitoes used for this study were physically transferred to the excreta-collecting funnel in the laboratory (adding another step), we envision (and have begun working toward) field-deployable devices of a similar principle that couple mosquito and excreta collection so that the virome of mosquito populations may be rapidly sampled without further mosquito processing. This will enable us to transition this funnel into contexts where it can actively expedite arbovirus surveillance and increase our capacity to monitor and respond to an ever-changing pathogen landscape. While such a device would allow for virus discovery and detection, drawbacks remain that must be addressed. Multiple mosquito species contributing to the excreta sample would not allow conclusive associations of viruses with hosts. Furthermore, even with the use of commonly employed mesh screening, small non-mosquito arthropods could potentially contaminate the sample. The core questions (in addition to virus discovery as performed here) that can be rapidly answered currently under these conditions focus on whether arboviruses are present at sites and locales, or with selective trap configurations that do not rely on broad insect attractants, such as light, and whether these arboviruses are present in mosquito species. These observations would, of course, be most powerful when virus-host associations are already known; however, when preceded by virus discovery, investigators can work backward, armed with the knowledge that particular pathogens are present. Field-deployed excreta sample collection tubes pre-loaded with nucleic acid preservation and/or lysis buffer to maximize sample integrity could allow surveillance to occur simply by removing and replacing the tube, rather than transferring mosquito catches to the lab, homogenizing, centrifuging, and moving forward. While qPCR is clearly advantageous for its ability to yield rapid results compared to shotgun metagenome sequencing and analysis, field-deployable microfluidic library preparation and sequencing devices are rapidly materializing, and an ultimate manifestation of this project could involve library prep, sequencing, and cellular data transfer occurring in the field at the trap itself.

## Data Availability

The raw Illumina sequence data generated in this study have been deposited in NCBI under BioProject PRJNA1417497 accessions SRX32046358, SRX32046357, and SRX32046356. Viral scaffolds discussed in the manuscript are provided in Table 1.

## SUPPLEMENTAL MATERIAL

Supplemental figure legend spectrum.00938-26-s0001.docx Descriptive legend for Fig. S1.

Supplemental table legends spectrum.00938-26-s0002.docx Descriptive legends for Tables S1 to S3.

Fig S1 spectrum.00938-26-s0003.pdf Maximum likelihood trypanosome 18S SSU phylogeny comprising SILVA 138.2 trypanosomatid taxa with non-redundant BLASTn top hit overlapped read pairs.

Table S1 spectrum.00938-26-s0004.xlsx Mosquito genomes queried along with the NCBI nt nucleotide database during the BLASTn homology search of RNA scaffolds assembled in this study.

Table S2 spectrum.00938-26-s0005.xlsx Detailed assembly information for each viral scaffold assembled in this study.

Table S3 spectrum.00938-26-s0006.xlsx BLASTn homology search output for all hits from merged paired-end reads to SILVA 138.2 Culicidae COI 5′ and 3′ references.

ASM does not own the copyrights to Supplemental Material that may be linked to, or accessed through, an article. The authors have granted ASM a non-exclusive, world-wide license to publish the Supplemental Material files. Please contact the corresponding author directly for reuse.
