# Supplementary material for: Metaviromic profiling of mosquito excreta using superhydrophobic collection devices expands the known RNA virome of North America
Source: Microbiol Spectr. 2026 Jul 28;14(9):e00938-26. doi: 10.1128/spectrum.00938-26 (PMC13532237; doi:10.1128/spectrum.00938-26)
Supplement: Supplementary file 1 [file spectrum.00938-26-s0003.pdf]

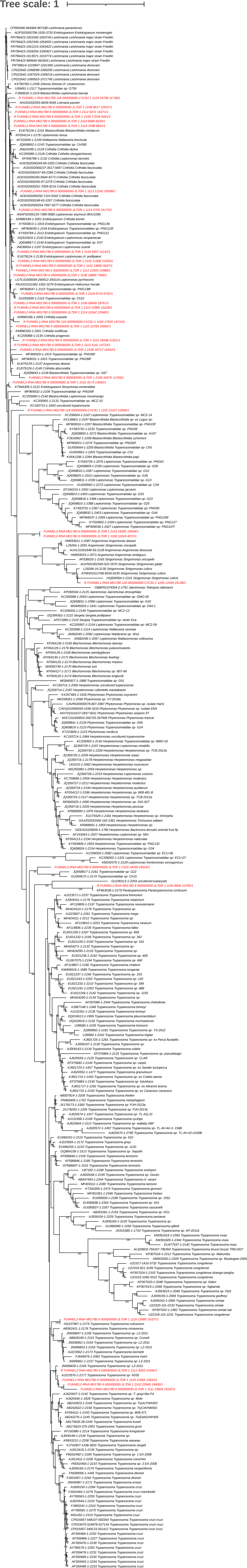
Tree scale: 1
