# Supplementary material for: Metaviromic profiling of mosquito excreta using superhydrophobic collection devices expands the known RNA virome of North America
Source: Microbiol Spectr. 2026 Jul 28;14(9):e00938-26. doi: 10.1128/spectrum.00938-26 (PMC13532237; doi:10.1128/spectrum.00938-26)
Supplement: Supplementary file 2 [file spectrum.00938-26-s0001.docx]

**Supplemental Figure Legends**

**Figure S1**. Maximum likelihood trypanosome 18S SSU phylogeny comprising SILVA 138.2 trypanosomatid taxa with non-redundant BLASTn top hit overlapped read pairs (red). The tree is manually rooted on Leishmania. Bootstrap supports > 90 are shown.
