# Supplementary material for: Metaviromic profiling of mosquito excreta using superhydrophobic collection devices expands the known RNA virome of North America
Source: Microbiol Spectr. 2026 Jul 28;14(9):e00938-26. doi: 10.1128/spectrum.00938-26 (PMC13532237; doi:10.1128/spectrum.00938-26)
Supplement: Supplementary file 3 [file spectrum.00938-26-s0002.docx]

**Supplemental Table Legends**

**Table S1**. Mosquito genomes queried along with the NCBI ‘nt’ nucleotide database during the BLASTn homology search of RNA scaffolds assembled in this study.

**Table S2**. Detailed assembly information for each scaffold assembled in this study. Column details are as follows: Baltimore class, Average fold coverage (BBmap), Length (nucleotides), Ratio of DIAMOND protein translation to length of NCBI reference, Ratio of scaffold nt length to BLASTn NCBI reference, Scaffold ID, P:{*} DIAMOND columnar protein homology output, N:{*} BLASTn columnar nucleotide homology output, BB:{*}BBmap output per-library mapping, NCBI organism assigned to top BLASTn hit.

**Table S3**. BLASTn homology search output for all hits from merged paired-end reads to SILVA 138.2 Culicidae COI 5’ and 3’ references.
